# Innovative strategies for a successful SLMTA country programme: The Rwanda story

**DOI:** 10.4102/ajlm.v3i2.217

**Published:** 2014-11-03

**Authors:** Innocent Nzabahimana, Sabin Sebasirimu, John B. Gatabazi, Emmanuel Ruzindana, Claver Kayobotsi, Mary K. Linde, Jean B. Mazarati, Edouard Ntagwabira, Janvier Serumondo, Georges A. Dahourou, Wangeci Gatei, Claude M. Muvunyi

**Affiliations:** 1 Rwanda Biomedical Center/National Reference Laboratory, Rwanda; 2 Rwanda Military Hospital, Rwanda; 3 Single Project Implementation Unit (SPIU)/Ministry of Health, Kenya; 4 American Society for Clinical Pathology (ASCP), United States; 5 US Centers for Disease Control and Prevention (CDC), Rwanda

## Abstract

**Background:**

In 2009, to improve the performance of laboratories and strengthen healthcare systems, the World Health Organization Regional Office for Africa (WHO AFRO) and partners launched two initiatives: a laboratory quality improvement programme called Strengthening Laboratory Management Toward Accreditation (SLMTA), and what is now called the Stepwise Laboratory Quality Improvement Process Towards Accreditation (SLIPTA).

**Objectives:**

This study describes the achievements of Rwandan laboratories four years after the introduction of SLMTA in the country, using the SLIPTA scoring system to measure laboratory progress.

**Methods:**

Three cohorts of five laboratories each were enrolled in the SLMTA programme in 2010, 2011 and 2013. The cohorts used SLMTA workshops, improvement projects, mentorship and quarterly performance-based financing incentives to accelerate laboratory quality improvement. Baseline, exit and follow-up audits were conducted over a two-year period from the time of enrolment. Audit scores were used to categorise laboratory quality on a scale of zero (< 55%) to five (95% – 100%) stars.

**Results:**

At baseline, 14 of the 15 laboratories received zero stars with the remaining laboratory receiving a two-star rating. At exit, five laboratories received one star, six received two stars and four received three stars. At the follow-up audit conducted in the first two cohorts approximately one year after exit, one laboratory scored two stars, five laboratories earned three stars and four laboratories, including the National Reference Laboratory, achieved four stars.

**Conclusion:**

Rwandan laboratories enrolled in SLMTA showed improvement in quality management systems. Sustaining the gains and further expansion of the SLMTA programme to meet country targets will require continued programme strengthening.

## Introduction

Reliable laboratory services are vital to a high-quality healthcare system; thus, investing in laboratory quality improvement is not only valuable, but essential.^1^ Despite a multitude of efforts to strengthen laboratories through infrastructure and human resource development, laboratory quality remains a challenge in resource-poor settings.^2,3^

Accreditation is a critical measure of a laboratory’s quality level, as recognised by a series of international conventions, which called for accreditation to be part of laboratory-strengthening efforts in low-income countries.^4,5,6,7^ In order to help address deficiencies in the system, two initiatives were launched concurrently in Kigali, Rwanda in July 2009 by the World Health Organization’s Regional Office for Africa (WHO AFRO) and partners.^4^ These were: Strengthening Laboratory Management Toward Accreditation (SLMTA), an innovative training and mentoring programme designed to facilitate the implementation of laboratory quality management systems in resource-limited settings;^8^ and an incremental laboratory accreditation preparation process, which later became known as the Stepwise Laboratory Quality Improvement Process Towards Accreditation (SLIPTA).^9^

Rwanda has a tiered laboratory system, funded through the Ministry of Health, which consists of the National Reference Laboratory (NRL) overseeing the entire laboratory network, four central referral laboratories, 43 district hospital laboratories and approximately 500 health centre laboratories. The NRL and five of the district hospital laboratories receive additional funding as part of the East African Public Health Laboratory Network (EAPHLN), a World Bank project aimed at controlling epidemics by strengthening laboratory capacity in five East African countries.

To date, Rwanda has enrolled 15 laboratories (three cohorts of five each) in the SLMTA programme. The Ministry of Health aims to eventually enrol all national, central and district hospital laboratories, a total of 48 countrywide, in the accreditation preparation process.^9^ This study describes the achievements of the first three cohorts of the SLMTA programme and shares their experiences and lessons learned four years after the launch of the programme in Rwanda.

## Research method and design

### SLMTA sites and training

In January 2010, the Rwandan Ministry of Health enrolled its NRL, three central referral laboratories (Centre Hospitalier Universitaire de Kigali [CHUK], Centre Hospitalier Universitaire de Butare [CHUB] and King Faisal Hospital [KFH]), as well as one military hospital, Kanombe Military Hospital (KMH), into the first cohort of SLMTA (Cohort I). Twenty-three participants were trained: three from CHUK (one laboratory manager and two laboratory technologists), three from CHUB (one laboratory manager and two laboratory technologists), three from KMH (one medical doctor in charge of paediatrics and two laboratory technologists), three from KFH (one laboratory manager and two heads of units) and 11 from the NRL (two laboratory managers and nine heads of different sections). During the nine-month programme, participants attended three SLMTA workshops and implemented assigned improvement projects.

The second SLMTA cohort (Cohort II) began in November 2011 with the five district hospital laboratories funded by the EAPHLN project: Byumba, Gihundwe, Gisenyi, Kibungo and Nyagatare. The training included 14 participants from these laboratories, three participants each from four laboratories (one lab manager, one quality officer and one safety officer) and two from Nyagatare Hospital Laboratory (one lab manager and one safety officer). In addition, six staff members from Cohort I laboratories participated (four from the NRL, one from CHUK and one from CHUB) because of a need to replace SLMTA-trained staff lost due to turnover and transfers.

In March 2013, five additional district hospital laboratories (Bushenge, Kibagabaga, Ruhango, Ruhengeri and Rwamagana) were enrolled in Cohort III. Each laboratory provided three participants: one laboratory manager, one quality officer and one safety officer. In addition to these 15 participants, laboratories from previous cohorts sent 11 participants (five from the NRL, two from KMH, one from CHUK, one from CHUB, one from Kibungo and one from Nyagatare), again to replace trained staff who had left.

### Audits

To evaluate progress, audits were conducted for all three cohorts using the SLIPTA checklist, before (baseline) and after (exit) SLMTA workshops. Depending on the audit scores, laboratories were awarded zero to five stars. A rating of zero stars was given for a score of < 55% (0–141 points ), one star for 55% – 64% (142–166 points), two stars for 65% – 74% (167–192 points ), three stars for 75% – 84% (193–218 points), four stars for 85% – 94% (219–243 points) and five stars for ≥ 95% (244–258 points).^10^ Follow-up audits (performed from three to 18 months after the exit audits) were conducted for Cohorts I and II, but follow-up audits for Cohort III laboratories had not yet been completed at the time of the writing of this article. Cohort I laboratories received one follow-up audit, with the exception of NRL, which had four. In Cohort II, Byumba, Gihundwe and Gisenyi each had two follow-up audits, whereas Gihundwe and Nyagatare had three.

All audits for Cohort I were conducted by consultants from the American Society for Clinical Pathology (ASCP). ASCP consultants teamed with Rwanda SLMTA facilitators to conduct baseline and exit audits for Cohorts II and III, whilst EAPHLN auditors conducted follow-up audits for Cohort II. The Ministry of Health selected two high-performing laboratories from Cohort II for official SLIPTA audit by the African Society for Laboratory Medicine (ASLM), namely, Nyagatare Hospital Laboratory and Gihundwe Hospital Laboratory.

### Mentorship and performance-based financing

Seventeen local mentors with advanced diplomas or bachelor’s degrees received a two-day training in-country in March 2012 in order to facilitate the implementation of quality management systems in the laboratories. They were tasked with helping SLMTA participants in the implementation of improvement projects, in reviewing lessons learned during workshops and in closing gaps identified during the audits. These local mentors visited each laboratory for five days following each workshop. Additionally, for Cohort II, mentors (two from Rwanda, one from Uganda) with Master’s degrees in microbiology spent two weeks per month in the laboratories from May 2012 to December 2013, overlapping with SLMTA implementation.

Cohort II laboratories also implemented performance-based financing, the first time such a model had been used with SLMTA. The performance-based financing model is a contractual approach stipulating that services and purchasing activities performed by health providers must be of good quality and compliant with standards. Linking financial incentives for the facility with results is designed to motivate healthcare providers to provide health services according to the qualities required by national norms and standards. A payment amount of $15 000 was allocated on a quarterly basis to each Cohort II laboratory with a score of 100% on the SLIPTA checklist. The incentive was discounted based on the SLIPTA audit score for laboratories not achieving a score of a 100%. For example, if a laboratory received a score of 70% on the SLIPTA checklist, it would receive a payment of 70% of $15 000, or $10 500. To incentivise continuous quality improvement, performance-based financing allowances were withheld if the laboratory’s SLIPTA score dropped by ≥ 3 percentage points from its previous score or resulted in a lower star rating. The laboratory could use this incentive money to buy commodities and conduct post-audit activities, gap analysis, workshops and employee-recognition activities.

## Results

### Cohort I

**FIGURE 1:**
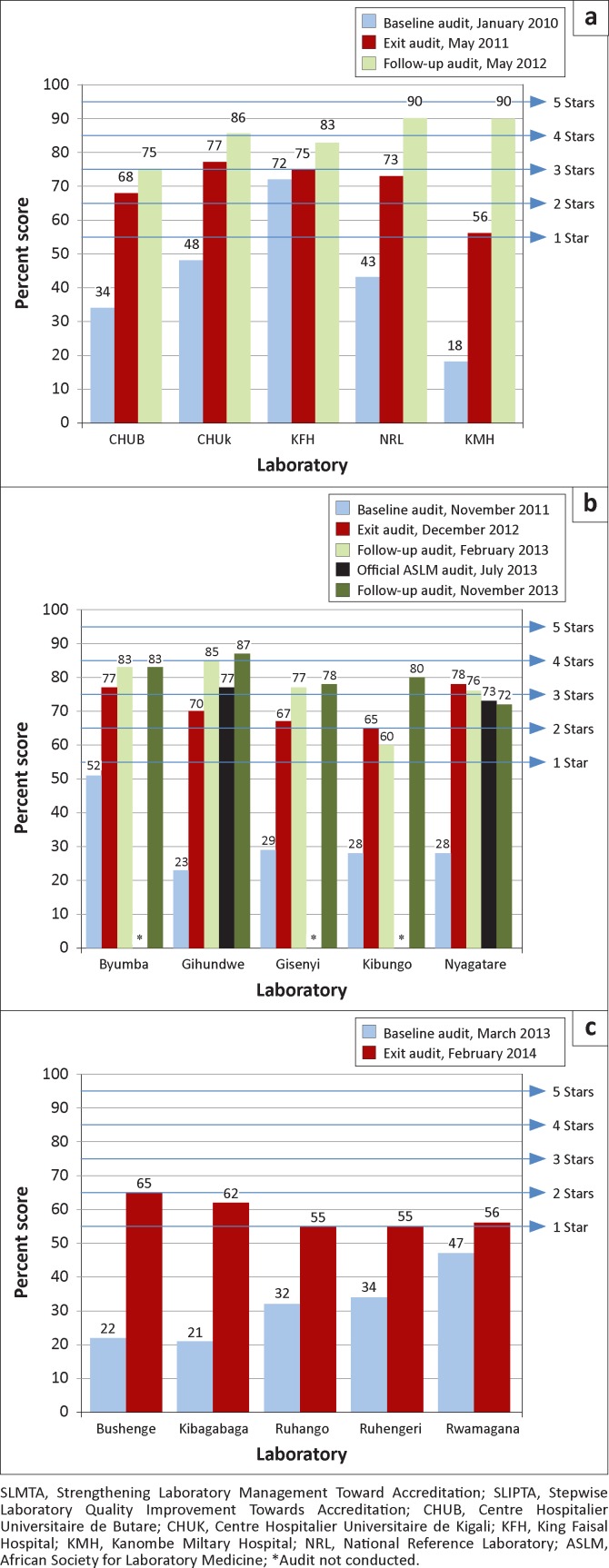
Progress of SLMTA Cohorts I (a), II (b) and III (c) in Rwanda based on SLIPTA checklist scores.

At the baseline audit for Cohort I, four laboratories had zero stars (CHUB, CHUK, NRL, KMH) and one laboratory (KFH) was at two stars (Figure 1a, Table 1). KFH is a private hospital laboratory and had been pursuing hospital accreditation actively for three years prior to enrolment in SLMTA. At the exit audit, one laboratory (KMH) received one star, two laboratories received two stars (CHUB, NRL) and two laboratories received three stars (CHUK, KFH). There was marked improvement in all laboratories, with median scores increasing from 43% to 73%. At the follow-up audit, one year after the exit audit, two laboratories earned three stars (CHUB, KFH) and three laboratories achieved four stars (CHUK, NRL, KMH).

**TABLE 1:** Cohort-level audit scores.

Cohort	Baseline audit		Exit audit		Median improvement from baseline to exit audit		1-year follow-up audit		Median improvement from exit to follow-up audit	
	Median %	Range	Median %	Range	Percentage Points	Range	Percentage Points	Range	Percentage Points	Range
**Cohort I**	43	18–72	73	56–77	30	3–38	86	75–90	9	7–34
**Cohort II**	28	23–52	70	65–78	38	25–50	80	72–87	11	6–17
**Cohort III**	32	21–47	56	55–65	23	9–43	-	-	-	-

### Cohort II

In Cohort II, all laboratories received zero stars at the baseline audit (Figure 1b, Table 1). At the exit audit, three laboratories received two stars (Gihundwe, Gisenyi, Kibungo) and two laboratories received three stars (Byumba, Nyagatare). Median scores increased from 28% at baseline to 70% at the exit audit. At the first follow-up audit, three months after exit, one laboratory was at one star (Kibungo), three laboratories had earned three stars (Byumba, Gisenyi, Nyagatare) and one had earned four stars (Gihundwe). At the official SLIPTA audit conducted by ASLM in July 2013, five months after the first follow-up audit, Nyagatare Hospital Laboratory was awarded two stars and Gihundwe Hospital Laboratory three stars. Scores were somewhat lower (three percentage points for Nyagatare Hospital Laboratory and eight for Gihundwe Hospital Laboratory) than those received at the first follow-up audit. A second follow-up audit in November 2013 resulted in similar scores to the first follow-up, with the exception of Kibungo Hospital Laboratory, whose score increased 20 percentage points to 80% (Figure 1b).

### Cohort III

At the baseline audit for Cohort III, all five district hospital laboratories had zero-star ratings (Figure 1c, Table 1). At the exit audit, four laboratories received one star (Kibagabaga, Ruhango, Ruhengeri, Rwamagana) and one laboratory received two stars (Bushenge). Median scores increased from 32% at baseline to 56% at exit.

### National reference laboratory

**FIGURE 2:**
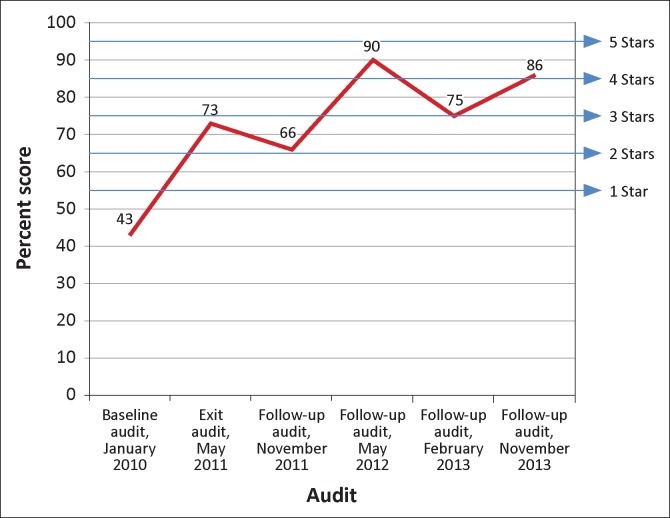
Progress of the National Reference Laboratory (NRL) from 2010–2013, based on Stepwise Laboratory Quality Improvement Toward Accreditation (SLIPTA) checklist scores.

The NRL participated in six audits during the period of 2010 to 2013. The laboratory showed marked, though unsteady, improvements from 43% at baseline to 86% at the fourth follow-up audit nearly four years later. At the first follow-up audit in November 2011, the NRL received two stars, a score similar to that awarded at the exit audit six months earlier. At the second follow-up audit in May 2012, the NRL earned four stars, but at the third follow-up audit in February 2013, the NRL decreased slightly to a three-star rating (Figure 2).

### Performance-based financing

Performance-based financing incentives of $75 000 were planned to be awarded to the five laboratories in Cohort II for each quarter. The maximum amount received in a quarter was $13 050 by Gihundwe laboratory which scored 87% at their first follow-up audit. Two laboratories (Nyagatare and Kibungo) were not awarded incentives for one quarter because of a drop in star levels.

## Discussion

Results of this study show substantial improvement in laboratories enrolled in SLMTA since 2010, as shown by star rating results. All but one of the 15 laboratories had a zero-star rating at the baseline audit, suggesting very low levels of quality management. At the conclusion of the SLMTA training programme, every laboratory had achieved at least one star, with four laboratories obtaining three or more stars. Furthermore, laboratories continued to improve after the end of the SLMTA programme, with nine of the 10 laboratories conducting follow-up audits achieving three or more stars.

Establishing a stepwise approach in order to guide laboratories in a gradual improvement process, as well as offering evaluations that demonstrate progress at each level, is a dynamic way of implementing quality laboratory standards in developing countries.^11^ Improvements resulting from SLMTA implementation have been observed elsewhere; however, Rwanda’s results are somewhat higher than what is typically found. For example, amongst 321 laboratories worldwide that have completed the SLMTA training, nearly one third (29%) remained at zero stars after SLMTA implementation, with a mean score increase of 23 percentage points, compared with Rwanda’s results of all laboratories achieving at least one star and a median improvement of 34 percentage points.^12^

For system-wide improvement, the Rwandan government encourages collecting and using laboratory data for advocacy; programmatic data are now used in developing policies aimed at improving quality services. For example, a cross-cutting problem in many laboratories in Rwanda has been service interruptions as a result of stockouts and equipment breakdowns. To address this problem, CHUK conducted an improvement project between its second and third SLMTA workshops which focused on calculating the financial impact of service interruption. From July to September 2010, stockouts and equipment breakdowns prevented the laboratory from performing 6486 tests, which were referred to private laboratories. The laboratory estimated that, if performed, the tests would have generated revenue of $14 308. In contrast, the funds needed to purchase the necessary reagents and maintain equipment were estimated at $5711, resulting in a net loss of $8597 in potential income to the hospital. After reviewing these findings, hospital senior management agreed to purchase a back-up clinical chemistry analyser and signed a maintenance agreement with laboratory equipment manufacturers with the aim of ensuring continuity of laboratory services.

Sustainability is a critical issue for SLMTA and other improvement programmes. Data from Cohorts I and II show that not only were the gains achieved through SLMTA implementation sustained a year after completion of the training programme, but they continued to increase a median of 10 additional percentage points. The KMH laboratory in Cohort I showed the greatest post-SLMTA improvement, with scores increasing from 56% (one star) at the exit audit to 90% (four stars) one year later. This laboratory had the lowest baseline score amongst all laboratories in Rwanda’s SLMTA programme to date, yet has now earned the highest follow-up score in the country’s programme. Staff at KMH attributed this remarkable achievement to high levels of commitment, team work and hospital management support of and direct involvement in the quality improvement effort. The KMH staff’s pride in their accomplishments is highlighted by the fact that in May 2012 they changed their name from Kanombe Military Hospital, which was linked to their military camp, to Rwanda Military Hospital (RMH). They also began to expand their testing capacity by introducing new services, including molecular biology, enzyme-linked immunosorbent assays and systematic bacteriology culture, as well as building a new laboratory infrastructure in their preparation to transition into a referral hospital.

Overall, Cohort II showed the greatest improvement of the three cohorts, with a median improvement of 38 percentage points from baseline to exit and an additional 11 percentage points a year later (Table 1). Several factors may help to explain these successes. Firstly, these laboratories received additional funding from the World Bank’s EAPHLN in order to support improvement projects and other elements of quality management systems, including building infrastructure and purchasing back-up equipment and safety items such as first aid kits, spill kits and eye wash stations. Secondly, these laboratories had the benefit of extensive on-site expert mentorship to assist with improvement projects and programme implementation. However, Cohort II was not without challenges. For example, Nyagatare Hospital Laboratory, which was one of the two laboratories audited by ASLM, lost their quality officer (September 2012) and laboratory manager (August 2013); despite sending replacements to be trained along with Cohort III laboratories, their scores declined steadily after the exit audit, dropping from 78% at exit to 72% at the second follow-up audit 11 months later (Figure 1b).

Cohort II also implemented an innovative performance-based financing incentive system. Performance-based financing has been used by many development organisations to ensure greater accountability and to improve the efficiency of funded programmes.^13^ Haiti was the first low-income country in which health service providers were remunerated according to their performance.^14^ In Cambodia, performance-based financing was applied to the public sector; despite promising results, however, it did not materialise into a national policy.^15^ Rwanda has been on the cutting edge of this approach, implementing performance-based financing in several sectors since 2002.^16,17,18^

NRL staff participated extensively in all three cohorts, as this laboratory is expected to provide leadership and guidance on quality management systems for Rwanda’s entire laboratory network. Also, as part of the EAPHLN, the NRL was in a unique position to monitor the progress and challenges of SLMTA implementation in the network laboratories.

Multiple factors may have contributed to variability in audit scores for NRL. As the country’s only national reference laboratory, the NRL provides a large proportion of services and routine testing in the country. This creates a heavy and fluctuating workload for the staff and the staff may not consistently prioritise quality improvement activities. Variability in scores could also reflect the senior management’s lack of focus on the accreditation preparation process. To overcome these challenges, there has been renewed commitment by senior management to focus on strengthening the laboratory systems at the NRL. In March 2013, a laboratory technical working group was launched with an accreditation subcommittee. The NRL is also undertaking extensive decentralisation to reduce routine testing and workloads, enrolling in external quality assessment programmes and supporting mentorship in all sections of the laboratory. The Rwanda Ministry of Health is forging ahead with its goal of implementing SLMTA in the remaining district hospital laboratories and ensuring that laboratories sustain momentum after programme completion by integrating continuous improvement into routine management.

### Conclusion

In Rwanda, laboratories enrolled in the SLMTA programme demonstrated measurable improvements. Performance-based financing, intensive monitoring and supplementary financial resources may have contributed to gains in Cohort II laboratories. Strengthening of an effective laboratory technical working group is needed to oversee the accreditation preparation process, mobilise resources and further develop the plan outlined by the Ministry of Health for long-term sustainability of quality laboratory systems. Expanding the use of performance-based financing to incentivise the quality improvement process in Rwanda may contribute to accreditation readiness.
